# Will my patients get their residence permit? A critical analysis of the ethical dilemmas involved in writing medical certificates for residence permits in France

**DOI:** 10.1186/s12910-020-00500-7

**Published:** 2020-07-13

**Authors:** Johann Cailhol, Marie-Christine Lebon, William Sherlaw

**Affiliations:** 1 Laboratoire Educations et Pratiques de Santé, Université Paris 13, 74 rue Marcel Cachin, Bobigny, France; 2 Infectious diseases unit, Avicenne teaching hospital, APHP, 125 route de Stalingrad, 93000 Bobigny, France; 3 Institut Convergences Migrations, Campus Condorcet, Hôtel à projets, 8 cours des Humanités, 93300 Aubervilliers, France; 4 Laboratoire d’Etudes et de Recherche en Sociologie, Université de Bretagne Occidentale, 20 rue Duquesne, 29200 Brest, France; 5 Ecole des Hautes Etudes en Santé Publique, 15 avenue du Professeur Léon-Bernard, 35043 Rennes, France

**Keywords:** France, Ill-migrants, Physicians, Ethical dilemma, Temporary residence permit

## Abstract

**Background:**

France has long been a country of immigration and in some respects may be seen to have a generous policy with respect to asylum seekers and access to health care for migrants. The French state notably provides healthcare access for undocumented migrants, through state medical aid and since 1998 has had a humanitarian policy for granting temporary residence permits for medical reason (TRPMR) to migrants. Within a context of political debate, reform and tightening immigration control we will examine this latter policy focusing especially on the dilemmas that arise for physicians of migrant patients when they are requested to write medical certificates as part of a TRPMR application. In a 2017 reform the key role of making recommendations on the granting or not of permits was handed over to Ministry of the Interior health inspectors. Recommendations are made after perusal of medical certificates established by the migrant’s physician and complementary evidence.

**Main body:**

The writing of medical certificates by a physician would seem straightforward. This is far from the case since it raises a number of ethical dilemmas. These occur within a physician-patient relationship embedded within a social contract between the State, the physician and the migrant patient. To clarify the ethical issues arising 3 vignettes based on practice within an infectious disease unit at a large Paris hospital have been developed. The vignettes highlight ethical dilemmas in the care for migrants with tuberculosis (dilemma in defining health and disease), chronic hepatitis (dilemma between beneficence and do not harm), and HIV / AIDS (issue of deservingness). We will go on to reflect on issues of social justice and responsibility for the health of migrants within a globalized world.

**Conclusions:**

Criteria for residence permit delivery appear less than clear-cut and are interpreted in a restrictive way. Neither are the consequences of refusing a residence permit taken into account. We call for an empirical transnational ethics study involving countries implementing similar TRPMR policies. We also call for inclusion of lobbying competences into the medical undergraduate curricula, in order to breed future generations of physicians skilled in defending social justice.

## Background

### The French policy context to migrant health

France has long been a country of immigration especially from its former colonies. It has also proudly proclaimed itself as a land of human rights and asylum and in some respects may be seen to have a generous policy with respect to asylum seekers in general and in particular the health of migrants. The French state notably provides health insurance cover and healthcare access for undocumented migrants, through state medical aid (AME Aide Médicale de l’Etat) [1]. It also has had a policy for granting temporary residence permits for medical reason (TRPMR) since 1998. This latter policy will be subject to deep scrutiny in this article in relation to the moral dilemmas that are raised for physicians. Nevertheless, immigration policy also can be seen in recent years, to favor restriction and strict control and thus maybe termed ambivalent. Drawing on Wolff’s public policy analysis^1^ it is useful to divide opinion on immigration into Cosmopolitan and Nationalist informed views ([2]): a) ‘Cosmopolitans’ argue *“that we are all equally citizens of the world, and political boundaries have no moral force”.* This is inspired by Kant’s view that humans have unalienable rights and dignity as persons, without reference to origin, current state, behavior or beliefs. Migrants like all other people have the right to health and the health care service has the obligation to treat them like all other people; b) Nationalists, to paraphrase Wolff, consider that the nation state has ‘the right to control its own territory, including who may reside within it.’ Each ‘country has developed its own culture which its citizens are likely to wish to see preserved, and they have built up the infrastructure through their efforts and taxes.’ As Wolff explains ‘being born into a political community as a rightful citizen (as distinct from some sort of visitor) creates a deep connection which generates a special set of rights and duties. No one else can have such rights and duties except under exceptional circumstances.’ In line with this it is easy to draw the conclusion that people from outside the country have less rights than those who are natives. Both sets of views may obviously impact on how people view health care for migrants. Now such views inform the political debate in France and are especially poignant in the wake of the different migrant crises notably in Calais [3] and Northern Paris and the increasing flow of migrants from across the Mediterranean [4]. Immigration has, of course, long been a controversial and divisive area of politics and has fueled the emergence of far-right populist political movements. In 2017, Marine le Pen from the far right Rassemblement National (former National Front) polled over a third of the votes and is the major rival to the current French President Emmanuel Macron [5]. Such trends have sorely tested the humanitarian strain to French policy. Macron’s government voted the « asylum and immigration law » in July 2018, aiming at strictly regulating and containing immigration [6], despite opposition from the National Consultative Human Rights Commission [7].

It is in this context of reform and immigration control that we wish to describe and analyze the ethical dilemmas which arise for physicians treating migrants who seek medical certificates to support their application for TRPMR, in order to stay or prolong their stay in France for health reasons.

### Consequences for migrants and conflicting roles for physicians

Obtaining a medical certificate to support an application to stay legally in France is crucial for a migrant’s future. Thus, the practice of establishing a medical certificate assumes great importance. In theory, this would seem straightforward, given that the physician is assumed to be neutral and objective in drawing up any certificate [8]. The reality is quite different, since the certificate writing occurs within a peculiar patient-physician relationship where the patient’s origin plays an uninvited role and where physicians’ opinions have the power to influence a migrant patient’s life, well beyond the health domain. Physicians completing forms are thus acutely aware of the consequences of rejection. They are prey to conflicting roles. On one hand they are acting as caring physicians in a trusting relationship with their patient and on the other they are called upon to assume an administrative role in the government immigration control system. This often leads to moral dilemmas which also may be compounded by the inherent uncertainties present in medical practice.

### Aims and plan of article

This article seeks to explore what is involved in such dilemmas through analyzing three vignettes drawn from clinical practice at a hospital infectious disease department in the northern Paris area. We will first describe the procedure how migrants may apply for TRPMRs. We will then go onto discuss the dilemmas inherent in each vignette. We will particularly focus on the physician-patient relationship and point out it may become a fundamentally asymmetric relationship when medical certificates are sought. To cast specific light on the duties and the diverse roles physicians are called to play we will present and adapt Cruess and Cruess social contract framework of the Patient –Physician relationship [9], to migrant health. In the light of this we will go onto further examine the multiple and contradictory expectations on physicians. We will highlight the impact on trust within the patient-physician relationship, due to difficulties involved in assuming the dual role of patient advocate and gatekeeper to social goods obtained through the granting of the TRPMR. We will conclude our investigation by first raising questions of social justice and responsibilities for the health of migrants in a globalized world and then briefly put forward suggestions for further research and recommendations relating to the issues raised.

### The TRPMR system

Temporary residence permit for medical reasons (TRPMR), a sub-category of humanitarian permits, exists in several countries in Europe [10], and was created in France in 1998 (Code d’Entrée et de Séjour des Etrangers et Demandeurs d’Asile article L.313–11). Patients are eligible for the granting of a TRPMR if, *inter alia*, “one’s state [of health] necessitates medical treatment, which in the event of its lack, could lead to extremely grave consequences for one’s health, and if such appropriate treatment cannot be accessed in one’s country of origin”(authors translation of [11]). The notion of “medical treatment”, is restrictively interpreted in the law, as a drug prescription.

TRPMR applications include a medical certificate, which is completed by the migrant’s physician. Until recently, Regional Health Agency Inspectors, physicians by training and acting independently from the Ministry of the Interior (MoI), examined applications and gave their recommendations. The Prefect, a local representative of MoI, made the final ruling on granting or not a permit. However, since January 1st 2017, a new reform was implemented and applications for TRPMR are now directly examined by physicians under contract of the Office Français pour l’Immigration et l’Intégration (OFII-French Office for immigration and integration-), a section of the MoI. This affiliation has removed the independence of physicians examining the applications [12–14].

**Fig. 1:**
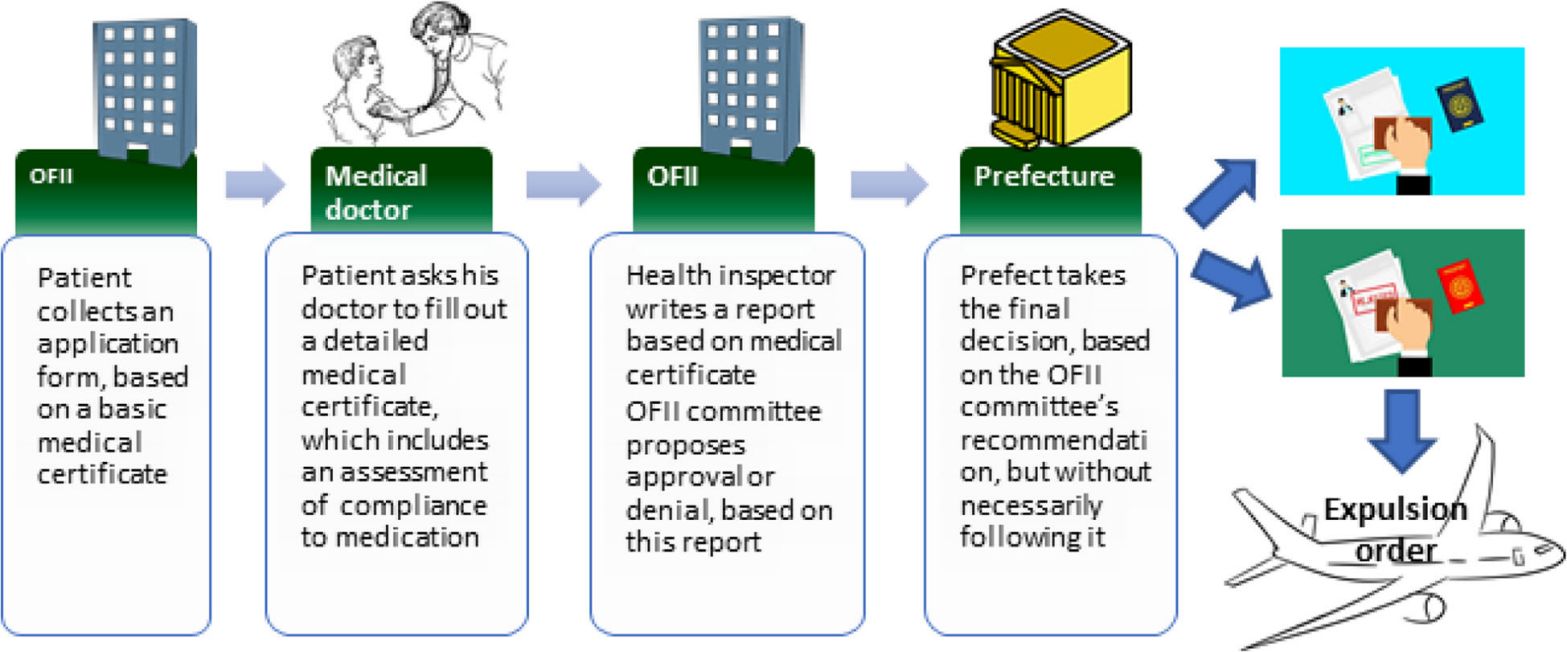
Application process for a temporary residence permit for medical reasons

The medical certificate, which must be included in TRPMR applications, describes in detail the health condition of the applicant, including “adherence” to medications. There is also a section for any “observations” that the physician may wish to make. Since January 1st 2017, there is no specific requirement relating to which physician should write the medical certificate, thus patients usually ask either their general practitioner, or hospital specialist to do this (see Fig. 1).

The repertoire of global access to medicine and to care (Bibliothèque d’Information Santé sur les Pays d’Origine- BISPO), established under the responsibility of MoI [15], is used as a guide by OFII health inspector when writing the report.

According to the 2017 OFII annual report, a total of 44,309 applications were processed, amongst which 45.4% were unsuccessful (compared to 23% in 2013, out of 26,026 applications).

Patients whose applications have been refused have three options: 1) to return to their home-country with a French government grant; 2) to appeal against the decision; 3) to apply for asylum (if they hadn’t previously applied). Once possibilities have been exhausted, any patient who wishes to stay in France will become an undocumented migrant, who still has the right to benefit from basic health care access (AME – Aide Médicale d’Etat). In practice though, they are likely to experience obstacles to accessing healthcare, and are under threat of deportation if arrested [16].

Almost 10% of total annual applications for TRPMR in France are processed in Seine-Saint-Denis, a deprived « Departement » comprised of 29% of migrants, in the North-Eastern outskirts of Paris [17]. Infectious diseases (such as complex tuberculosis, HIV, hepatitis B or C) together with mental health conditions (such as post-traumatic stress disorders or severe depressions) are the most frequent conditions represented [18].

### Understanding the ethical dilemmas raised by TRPMR through vignettes

At Avicenne University hospital, located in Seine Saint Denis Departement, physicians in the infectious disease unit are much used to writing such medical certificates (some 200 certificates per year are issued by the unit). Following refusal of a residence permit application, patients, naturally enough, seek help from their physician who wrote the certificate in the first place. This often leads to physicians experiencing a dilemma: how may they care for these patients they are responsible for, to the best of their abilities, while respecting their legal and professional obligations in particular, as stated in the physician charter [19]. More broadly it poses the question of what their moral duties as a physician and as a citizen are, in this peculiar context. We may mention briefly here that the historical legacy of World War 2 renders French physicians, as citizens, particularly sensitive to issues with respect to informing authorities about migrant patients in their care.^2^ In order to highlight such issues 3 vignettes were drawn up. These all feature infectious diseases since these are the commonest conditions cited in TRPMR. The vignettes may be considered to be representative of practice at the Avicenne hospital infectious disease unit.

## Main text

### Vignette 1: pertaining to definitions of disease and health

An undocumented migrant diagnosed with pulmonary and pleural tuberculosis (TB) was provided with a TRPMR. Following successful treatment and cure a year later a renewal of his TRPMR was sought. Another medical certificate was delivered, stating that his status needed to be consolidated, and another year of TRPMR was delivered. A year passed and the patient came back to ask for another certificate, but there were no further medical reasons to write a certificate. The patient did not present any symptom of TB relapse. The physician did not write the certificate, informing the patient there was no reason for writing it. Should a physician write a false certificate, the risk is to be suspended from the Physician’s order, to lose one’s license to practice medicine as well as a heavy fine, as stipulated in the Code of Deontology [7]. The patient came back some months later with an expulsion order.

If one were to analyze this situation only through a “disease” lens, indeed, no justification exists to certify that the patient had a severe disorder, which, if left untreated, would lead to a grave risk to health. However, let’s now look at consequences of this patient being denied a residence permit. The patient had 2 options left. The first option would be to leave France, with some financial aid, in order to re-settle in his country of origin, Mali, which he left 8 years earlier. He had built a significant social network in France, and would lose its benefit when sent back, and he would also possibly be exposed to stigmatization and a deterioration of mental health. Such consequences of ‘forced return’ have been described by Kone et al [21]. The second option would be to stay in France, despite being served with an expulsion order, and thus become an undocumented migrant. As stated by the European Agency for Fundamental Rights [22], this illegal status also exposes the patient, *inter alia*, to a range of mental health disorders, due to the high degree of anxiety associated with this status. Furthermore undocumented status increases the risk of contracting or reactivating TB [23]. Hence, both returning to their country of origin (forcedly) or staying undocumented may have a profound negative effect on migrants’ health. Evidence is lacking on the outcome of being sent back. Indeed, as responsibilities stop at the geographical frontier, there is no follow-up of migrants’ health status once sent back. Professional responsibility includes hypothesizing different *scenarii* for patient’s health outcome, however.

The issue being highlighted here is what should be assessed by the medical certificate? Should physicians be asked to solely and narrowly assess the risk of a given disease worsening if the patient were sent back to their country of origin (current model of certificate)? Or should physicians be allowed to take into account a significant impact of being denied a permit, on the migrant’s health, from a holistic point of view, such as put forward by Engel [24], Nordenfelt [25] and more recently Ventakapuram [26], who espouse holistic and biopsychosocial models of health. Such models would underpin models of care which take into account non-biological determinants of health. The medical certificate model, as it stands today, does not offer space for such description nor assessment of a patient. In a similar vein, Fassin once noticed a shift from asylum applications to TRPMR applications, as success rates to asylum applications fell. In relation to this, Fassin has developed the notion of biological body by opposition to social body. He highly criticized the overemphasis on categorizing humans as merely diseased objects, based on biological parameters and associated measurements which severs human narratives and relationship [27]. The least physicians could do it to use the tiny space allocated to “remarks” to describe broader psychosocial consequences of denying a permit, in order to counteract the current biomedical dogma on which the TRPMR application form is based.

### Vignette 2: pertaining to severity and line-drawing

A woman from the Ivory Coast, diagnosed with hepatitis B, not yet meeting the antiviral treatment criteria, asks for a certificate. Clinical guidelines in terms of hepatitis B evolve quickly, and time to start treatment depends on biological criteria, which may fluctuate over time [28]. Therefore, patients need to be monitored over the long term. Consequences of chronic hepatitis B can be severe, if monitoring is insufficient, and timely treatment is not given, with progression to cirrhosis and liver cancer.

In the case where patients are left untreated and only monitored (as in this vignette), they are usually not entitled to receiving a TRPMR since the certificate considers only the present time. The administrative timescale is frozen and does not correspond to the timescale of illness. When physicians write the certificate, they have in mind the fact that the application might not be successful [29]. Even though physicians have no duty to assess whether their certificate will be successful or not, they cannot erase this from their mind, since they are not only a technical expert, but also clinicians under Hippocratic Oath. The physician thus faces an ethical dilemma: either to introduce a treatment with potential side effects, which would certainly allow the patient to get a TRPMR; or to respect current guidelines and leave the patient to be sent back to her country of origin. To actively prescribe a treatment which is not *strictosensu* indicated is a far more litigious decision, than not to prescribe a treatment in a patient who in theory needs it. In terms of bioethics principles, the principle of non-maleficence (to not prescribe a treatment when not needed) might temper the one of beneficence (to prescribe a treatment in order to favor the residence permit) [30].

Guidelines for treatment of a disease may evolve as new evidence emerge. Therefore, categorization of a patient as diseased or healthy might also vary over time, and the patient may change from being categorized as an inactive hepatitis B carrier, into being categorized as being an active hepatitis B carrier requiring rapid treatment. Where should the line be drawn? How can we measure the severity of a disease, when its evolution is indeterminate, without taking into account the context, while at the same time severity is the door opener to obtain a residence permit? Here the notion of uncertainty or indetermination in disease definition would seem to be of crucial importance [31].

### Vignette 3: pertaining to deservingness

A man was diagnosed in 2001 for Human Immunodeficiency Virus (HIV) and put on antiretrovirals. However, his adherence was low, and he never reached undetectability. He never agreed to disclose his HIV status to his spouse. Eventually, years later, his spouse was diagnosed with HIV at a very advanced stage, and started her follow-up at the same clinic. From her history taking, it emerged that she had tested HIV positive some years earlier, but her husband had prevented her from getting the results (there is no mandatory tracing / reporting of contacts in France). She declared she had never had any other partner besides her husband. Thus, it was obvious that her husband was the unique source of transmission. The husband returned to the clinic to get a medical certificate, in order to apply for a TRPMR. How should the physician react bearing in mind that the patient had deliberately obstructed timely care for his spouse? Might there be a certain reluctance to fill in the application form?

The concept underpinning this case is deservingness. In clinical medicine it is generally held that care should be given according to clinical criteria only, while not taking into account physician’s moral judgements [32]. To take into account a patient’ social worth or past behavior potentially related to a current diagnosis should be out of question. However, this issue has been well explored in the case of liver grafts, and deservingness in reality may often enter into account when making clinical choice [33]. In the same way, if physicians do not think that the patient deserves to stay in France, they may be tempted to write the certificate in a way to decrease a patient’s chances to obtain a permit.

This vignette may raise even more acutely felt dilemmas than those experienced in the previous cases. On one hand, writing the certificate without mentioning the poor adherence to treatment will allow him to stay in France and get continuous care. On the other hand, mentioning poor adherence may increase the likelihood of the patient being denied the permit. Staying neutral in this particular case is virtually impossible, since one knows the consequences associated with what one writes.

The “adherence” section in the certificate indeed serves to identify patients who are non- or poorly adherent. The lack of adherence, in principle, implies that patients do not deserve the permit from an administrative perspective (personal communication from OFII officer). However, adherence to treatment is difficult to determine. Nevertheless, the fact of being adherent to a treatment would seem to be integral to the tacit contract between the state and the patient being granted a TRPMR.

Given these issues, we wonder if the State is entitled to deny a TRPMR based on adherence information. What is the role of OFII in clarifying the terms of contract when delivering the permit?

Analysis of the 3 vignettes has highlighted a number of issues. These, although interrelated, may be roughly divided into: a) epistemological questions such as the definition of health itself, which may be viewed narrowly or more holistically; and the issues of so-called line-drawing with respect to disease severity assessment; b) moral questions, such as the judgement over deservingness and its effect on the process. The many different issues raised by our vignettes may be examined through different ethical prisms such as consequentialism examining the results of acts and decisions. They also may be examined through non-consequentialism through reference to Kantian inspired duties and obligations and their relationship to trust [34] as well as through the 4 well-known principles of bioethics, beneficence, non-maleficence, autonomy and justice [35, 36]. Arguably 3 of these especially (beneficence, non-maleficence and justice) are common to the Hippocratic Oath and the respect and consideration of autonomy of patients is upheld as a key feature of the ethical practice of modern medicine. Rather than explicitly favour one particular framework such as the principalism of Beauchamp & Childress [37] which may not provide all the answers, we have drawn on explicitly and implicitly a diversity of ethical tools. The issues raised by the vignettes may also be approached through virtue ethics to ask the important question “How should a physician act virtuously” [38]? What are the obligations of physicians towards different parties (i.e. patient, society, professional body), when dealing with the health of migrant patients?

### The patient-physician relationship

According to Cruess and Cruess, in modern healthcare systems, traditional bilateral patient-physician relationships should be seen as embedded within a wider set of reciprocal expectations and obligations involving different actors [9]. Such actors comprise government, health institutions, professional bodies, the legal framework, policy makers, the media, patients and citizens [9]. These come together to constitute what may be conceptualized as being a social contract [9].

We have adapted the framework to explore physician-migrant relationship in general and to specifically understand expectations and obligations involved in drawing up a medical certificate (Table 1).

**Table 1:** Reciprocical expections involved in the writing of medical certificates to apply for temporary residence permit for medical reasons, adapted from Cruess and Cruess [9]

	Expectations
Migrant patients of physicians	To be cured / to receive care / to be helped Altruistic service, beneficence Morality, integrity, honesty Trustworthiness
Physicians of migrant patients	To be trusted Rewards (non-financial – e.g. maintaining a relationship, gratitude-, financial) Adherence to care
Physicians of governments	Health care system: equitable, value-laden, reasonable freedom within the system To ensure health care access and continuity via delivery of health care permit
Migrant patients of governments	Equitable, trustworthy and accessible health care system To get residence permit for health care To not violate human rights
Government of migrant patients	Appropriate use of resources (referring to the social justice principle) Strict compliance to care Control overflow of migrants
Government of physicians	Morality, integrity, honesty Compliance with laws (i.e. providing objective assessment of health status) Promotion of public goods

### Multiple and contradictory expectations on physicians

Many expectations in Table 1 may be seen to be in tension or even conflicting. The most obvious example is the government expectation to reduce the number of unwanted migrants (by contrast to selected – [39]), which is very much in conflict with migrant expectations of getting a residence permit. Other obvious sources of tension are: government expecting physicians to comply with the law and assessing migrants’ health objectively, migrants expecting to be helped through getting a medical certificate and physician expecting a reward from the migrant (either moral or by maintaining a relationship), without infringing the law.

In addition to others having expectations of physicians, physicians have moral or professional obligations themselves. We will focus hereafter only on the physician perspective (expectations and obligations). On one hand, physicians have the moral obligations to protect the vulnerable [32]. These obligations, relating to beneficence and non-maleficence are the keystone of the Hippocratic oath [32]. Furthermore, obtaining a residence permit is arguably an important social factor which may favor patient health, and another clause of the Hippocratic oath relates to the protection of health, including in its social dimension [32].^3^ Obtaining a TRPMR could also be interpreted as a social right, to which physicians must facilitate access, in line with their professional code of deontology conduct [8]. On the other hand, physicians have legal obligation in terms of writing a medical certificate to include only facts based on their observations. A bogus certificate is a criminal offense, and is engraved in the professional code of conduct [8]. However, as is well-known, legal and moral obligations may lead to paradoxical injunctions (double binds) at times within health-care practice [40, 41].

On top of these, physicians are subject to external influences with regards to their perceptions of migrants. Such multiple-level influences are diffused through media channels, each of which having their own particular political bias. Physicians themselves have political views which may also influence their a priori perception of migrants and their degree of deservingness. Non-Governmental Organizations (NGOs) such as Comede or Gisti have been historically involved in human rights actions towards migrants and have been critical of the implementation of TRPMR policy. These NGOs suggest that OFII inspectors may be considered as answering to the MoI, thereby prioritizing immigration control, rather than having the individual migrant’s health as a priority [12, 13, 42]. All these influences will permeate the patient-physician relationship.

In our analysis, in all 3 vignettes, the principle of beneficence would demand that physicians protect the vulnerable by writing a certificate that would allow the patient to obtain the TRPMR. The MoI, via the intermediary of OFII, expects physicians to write an “objective” certificate, stating whether patients need a treatment the day the patient was examined, and how severe would the consequences be, if patients were to be sent back to their country of origin. Furthermore, a whole raft of supplementary obligations impinges on physicians. They are, of course, within the legal framework, expected not to write a bogus certificate, exaggerating the severity of a disease. They are also expected, in line with professional codes of practice [8], to follow the current clinical guidelines. Furthermore, the government expects them in relation to their professional responsibility, to protect public goods (i.e. national health insurance funds), public health (i.e. not expose citizens to HIV transmission from untreated individual) and wider common goods.

**Fig. 2:**
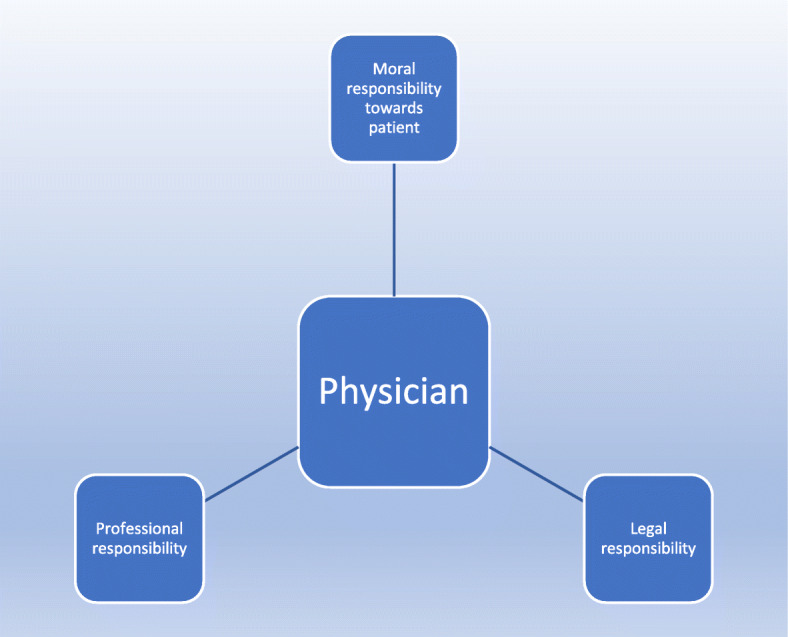
The inner world of the physician in a relationship with a patient

Hence, when writing a medical certificate for a migrant, physicians may face ethical dilemmas. They are caught between moral and legal expectations, while at the same time they are called upon to respect an implicit social contract, within a particular political context. These tensions are illustrated in Fig. 2.

### The difficult dual role of the physician: patient advocate and gatekeeper

Despite contradictory discourse from government and NGOs, in parallel to increasing rates of refusals of permits over the past months, there seem to be greater enforcement of expulsion orders [42, 43], reinforcing pre-existing suspicion with respect to the role of OFII.

One of the criteria examined for TRPMR is the probability of death or serious adverse effects occurring in the future. While this risk is a probability in the future at the population level, taken at the individual level, the risk is rather an uncertainty, which one day may become a reality. Patients who are sent back to their country would not be necessarily able to access adequate care. Indeed, effective access to care in a given country depends on multiple factors that go beyond patient and physician assessment, and concerns have been voiced about the reliability of an instrument such as BISPO, which is moreover sponsored by MoI.

These uncertainties are deeply anchored in physician’s mind when writing the certificate, for physicians care for unique individuals and not for data.

Given the above context, even if on paper the role of physicians is to offer technical expertise, other core values come to the fore. We may highlight professional virtue and the role of being an empathic compassionate caregiver [44–46]. Thus, a physician in line with the essential core goods of medicine may embrace the role of patient’s advocate in order to achieve the highest benefit for the migrant patient. Physicians are constantly called upon to balance between their roles of health advocate and gatekeeper to social goods opened up through the granting of a permit.

These public goods may be viewed as being under threat (or not) from the further arrival of new immigrants. The view adopted will depend on the political sensibility and knowledge of the physician, who may or not accept policy research evidence (e.g. suggesting that migrant health care - AME - represents an extremely small cost, and may indeed avoid incurring much larger costs for the health insurance system [1]).

Since physicians are increasingly impinged by a series of paradoxical injunctions (double-binds) [47] their position may become unbearable as the social and political context deteriorates.

This phenomenon has been observed previously with other types of certificate, such as those to obtain disability allowance or sickness leave. Physicians felt that the most difficult challenges were ethical dilemmas rising from tensions between their dual roles of « patient advocate » and « medical expert » [48–51]. The definitive nature of the denial or acceptance of a permit may make physicians feel that they are being cast in the role of judges passing sentence. In migrant health this is even harder to handle than ruling against granting disability allowance, since there is no possibility of re-application.

### Ethical care under threat in a globalized world?

The foundation of the physician-patient relationship is mutual trust. But on occasions, when the sick migrant asks for a medical certificate in order to apply for a health care permit, the relationship may become distorted, and a breach of trust appears. Illness might sometimes be characterized, and sometimes not. Physicians may think that, as obtaining the certificate is their aim, patients may desire to have a disease, unconsciously or not, influencing the patients' discourse. The quality of the patient-physician relationship will influence the level of trust physicians have in their patients’ discourse, which in turn will influence their interpretation of patients’ symptoms. Let us examine some possibilities. A patient complains of feeling diffuse pain everywhere: this is similar to when he was suffering from tuberculosis. However examination shows he is cured, so could this be explained by the fact he is ‘merely’ recalling his pain? Studies suggest that the memory of pain may be considered to be equivalent to actual pain [52]. Could a patient deliberately fail to adhere to treatment in order NOT to get cured, thus achieving ‘perpetual’ renewals of health permits, despite obvious risks for his/her own health? Could migrants deliberately put themselves at risk of contracting HIV in order to get a permit? As one migrant said: “I wish I had HIV, since only having hepatitis B does not allow me to stay”.

The fact that one is treating a migrant rather than a native patient of a country should not lead to differences in clinical management. However, since physicians are asked, between the lines, to assess the deservingness of migrants to stay, this may in fact occur. It would seem that the writing of certificates for migrant patients introduces considerable tension within the physician-patient relationship built upon mutual trust and may pervert the very essence of medical practice.

Finally, in a globalized world, who holds responsibility for the consequences of sending patients back or denying a permit, and casting them into undocumented status?

Is it the physician who feels great responsibility and inevitably poses the question about how they write their report? Is it OFII? The Prefect? The French government? The government of the country of origin who fails to provide appropriate care, and who has failed initially to retain their citizens within their homeland? Will one ever know what has happened to patients who were sent back? The responsibilities of states who send patients back seem to vanish once the patients cross the border, or if their status becomes undocumented, whereas, globally, their right to health stays unchanged. Rights to health do not stop at the borders of a state nor does citizenship. Signed and ratified conventions on human rights declare that everyone has “a right to the highest attainable standard of health” [53]. The rights of migrants to health would seem better described as having a right to a minimum standard of health. This really questions the global boundaries of legal responsibilities and suggests rather the need to apply a global health ethics approach to migrant health [54].

## Conclusions

We have described some of the ethical dilemmas which may be posed to physicians when writing a certificate for TRPMR application, due to a narrow interpretation of health, currently restricted to measurable disease [27, 55].

Implementation of a category of humanitarian permit, such as the TRPMR, is of crucial importance. However, in an era of enforced immigration control, the permit sits uneasily between the blurred boundaries of immigration control and humanitarian policies.

Should physicians be placed under quasi-unbearable tensions or forced to make radical choices, influenced by their own values, and virtue which honor the essential nature of caring and medical practice? With respect to TRPMR should physicians accept to become an instrument of the State [56]? Humans by nature are biased [57], and it is unreasonable to expect to stay neutral in face of dilemmas. Ultimately the inherent contradictions of a system come to be played out in the physicians’ treatment room leading to intractable dilemmas. On one hand government policy through its MoI aims to control and limit migration, while on the other it is responsible also for offering high standard healthcare for humanitarian reasons which, contrary to the previous arm of policy, tends to increase and welcome migrants to France. This contradiction is further exacerbated through different ways of considering health (and disease) and indeed standards of health on offer.

Amartya Sen, defines health as “physical and psychological status which allows for the full development of each person’ capabilities” (pp.965 in [58]). As Willen and *al.* advance, such a definition through integrating migration as a health determinant *per se* [59] would provide an egalitarian way of practicing migrant care [58].

In Belgium also, NGOs and physicians have started to raise their voices against restricted access to TRPMR [60]. There is an urgent need for empirical ethical studies, across countries which have similar laws, in order to gain further insights and influence policies. Such studies may include surveys, qualitative semi-directive and non-directive interviews, and focus groups with different stakeholders. It is also important to ask professional societies of physicians and its different ruling bodies in France to examine and offer their opinions on the issues we have raised.^4^ Analyzing physicians’ strategies in dealing with dilemmas arisen by migrants’ care would help to build best practice. This could be used as evidence to lobby legislators at the European level. Role of physicians as a legitimate defender and advocate of social justice, e.g. physicians advocating for better healthcare policies for disadvantaged populations, could also be included in undergraduate curricula. A good example to follow currently features in medical curricula in Canada [61]. Finally we would like to raise an additional question which has emerged through this study: does the scope of beneficence vary according to physicians’ values and models of health and care?

## Data Availability

Not applicable.

## Abbreviations

AME: Aide Médicale d’Etat
BISPO: Bibliothèque d’Information Santé sur les Pays d’Origine
OFII: Office Français de l’immigration et l’intégration
HIV: Human Immunodeficiency Virus
MoI: Ministry of Interior
NGO: Non-Governmental organization
TB: Tuberculosis
TRPMR: Temporary residence permit for medical reasons

## Footnotes

1 It is important to note that Wolff believes that ‘neither of these views can be dismissed out of hand’ and his aim is not to ‘defend cosmopolitanism or (liberal) nationalism’ Rather it is to understand the effect these views have on the debate on immigration’

2 This *general impression is supported by leading French philosophical and judicial commentators, André Comte-Sponville and Henri Leclerc. We translate here a few lines from a 2005 interview they gave on the subject of denunciation* [20]. **Henri. Leclerc**: Denunciation is far from being considered a citizen’s duty, it is tainted with connotations which have their roots in childhood. That starts at school. When we were children the tell-tale was punished by the school master. And then do you recall the novel ‘Mateo Falcone’ by Prosper Mérimée? A Corsican shoots his son in the head because he denounced a thief who stole watches! And don’t forget either the first article of the Universal Declaration of Human Rights which states “All humans should act towards each other in a spirit of fraternity.” That is why informing is so frowned upon. **André Comte Sponville:** We are deeply marked by the traumatic episode of the [German] Occupation. There were so many denunciations of members of the Resistance, of Jews, of clandestine people to the Gestapo or the Vichy authorities, that the very idea of denunciation became suspect. Contrary to popular belief, it is not denouncing which is inherent to informing, it is the underlying motivation of hate, ill-gotten gain, and selfishness. (Our translation)

3 The English and French versions of the Hippocratic Oath differ slightly, reflecting cultural differences. The French version is more abstract in terms of “social dimension of health”.

4 we kindly offer our thanks for this suggestion to one of our referees.
